# Diagnostic Challenges in Takotsubo Syndrome: Bridging Mimics, Mechanisms, and Management

**DOI:** 10.3390/jcm15135088

**Published:** 2026-06-30

**Authors:** Andreas Mitsis, Elina Khattab, Evi Christodoulou, Stefanos Sakellaropoulos, Nikolaos P. E. Kadoglou

**Affiliations:** 1 Cardiology Department, Nicosia General Hospital, State Health Services Organization, Nicosia 2029, Cyprus; khattab_elina@outlook.com; 2 Cardiology Department, Limassol General Hospital, State Health Services Organization, Limassol 4131, Cyprus; evi.christodoulou@yahoo.com; 3 Department of Internal Medicine, Cardiology Clinic, Kantonsspital Baden, 5404 Baden, Switzerland; stefanos986@hotmail.com; 4 Medical School, University of Cyprus, Nicosia 2115, Cyprus; nikoskad@yahoo.com

**Keywords:** acute coronary syndrome mimics, cardiac imaging, diagnostic criteria, differential diagnosis, InterTAK Diagnostic Score, stress-induced cardiomyopathy, takotsubo syndrome

## Abstract

Takotsubo syndrome (TTS), also known as stress-induced cardiomyopathy, is a form of transient left ventricular systolic dysfunction that typically mimics acute coronary syndrome (ACS). Although increasingly recognized, its diagnosis remains challenging due to heterogeneous clinical presentations, evolving pathophysiological concepts, and significant overlap with other acute cardiac conditions. Contemporary criteria, including the InterTAK diagnostic framework, aim to refine case identification, yet distinctions from myocardial infarction, myocarditis, and other cardiomyopathies often remain blurred. Advances in multimodal imaging, biomarkers, and artificial intelligence hold promise for improving diagnostic precision. This review explores current diagnostic challenges in TTS, integrating clinical presentation, mechanistic understanding, and management implications. By bridging mimics, mechanisms, and management, we highlight the need for a nuanced, multidisciplinary approach that balances clinical vigilance with emerging diagnostic tools to optimize patients’ outcomes.

## 1. Introduction

Since its first description in Japan in 1990, Takotsubo syndrome (TTS) has transitioned from a rare clinical observation to a well-recognized acute cardiac condition characterized by transient left ventricular (LV) dysfunction in the absence of obstructive coronary artery disease (CAD) [1]. Typically affecting postmenopausal women and often triggered by intense emotional or physical stress, TTS presents with chest pain, electrocardiographic (ECG) changes, and troponin elevation that closely resemble acute coronary syndrome (ACS). This striking similarity frequently leads to diagnostic confusion in emergency care settings. Despite growing clinical awareness, the diagnosis of TTS remains a challenge. Its pathophysiology extends beyond a simple catecholamine surge to include microvascular dysfunction, neurogenic injury, and autonomic dysregulation [2]. Furthermore, the increasing recognition of atypical forms—mid-ventricular, basal, focal, and global—has complicated the diagnostic landscape. Standardized criteria such as the InterTAK Diagnostic Score and multimodal imaging have enhanced specificity, yet the distinction between TTS and other transient cardiac-related syndromes, including myocarditis and ischemic stunning, remains difficult in practice [3].

This review explores the diagnostic challenges of TTS, bridging mimics, mechanisms, and management. We aim to outline the evolution of diagnostic criteria, identify common pitfalls in differential diagnosis, summarize key imaging and biomarker tools, and highlight future directions toward a more precise and integrative diagnostic approach.

### Methods and Search Strategy

This article was designed as a narrative review aiming to summarize contemporary diagnostic challenges in TTS, with an emphasis on clinical mimics, evolving diagnostic criteria, multimodality imaging, biomarkers, risk stratification, and management implications. A literature search was performed using PubMed/MEDLINE and Scopus for articles published up to May 2026. Search terms included “Takotsubo syndrome”, “stress cardiomyopathy”, “InterTAK Diagnostic Score”, “InterTAK Prognostic Score”, “acute coronary syndrome”, “MINOCA”, “myocarditis”, “cardiac magnetic resonance”, “echocardiography”, “coronary CT angiography”, “nuclear imaging”, “biomarkers”, “microRNA”, “copeptin”, “artificial intelligence”, and “management”. Additional relevant articles were identified from reference lists of key reviews, consensus documents, and registry studies. Priority was given to international consensus documents, registry-based studies, systematic reviews, meta-analyses, and clinically relevant observational studies. Because this was a narrative review, no formal systematic review protocol, risk-of-bias assessment, or quantitative meta-analysis was performed.

## 2. Evolving Diagnostic Criteria

The diagnostic framework for TTS has evolved from the restrictive Mayo Clinic criteria toward broader contemporary definitions (see Table 1). The original Mayo Clinic criteria were valuable for early standardization [4], but their strict requirement for the absence of obstructive CAD and exclusion of pheochromocytoma may under-recognize cases with concomitant CAD [5], atypical phenotypes, secondary triggers [6], or RV involvement [7]. The HFA/ESC position statement and the InterTAK diagnostic criteria therefore expanded the concept by recognizing emotional, physical, neurological, psychiatric, and endocrine triggers, atypical ventricular patterns, RV involvement, and coexistence with CAD.

A further diagnostic challenge is the recognition of atypical phenotypes. Although apical ballooning is the classical and most frequent phenotype [12], mid-ventricular, basal or “reverse”, focal, and biventricular forms of ventricular dysfunction [13] are increasingly recognized. These variants may be misclassified as ACS, myocarditis, or myocardial infarction with non-obstructive coronary arteries (MINOCA) if clinicians expect only the classical apical pattern. Focal TTS can closely mimic a regional infarction, while basal TTS may be confused with myocarditis, catecholamine-mediated injury, or neurogenic myocardial stunning [14]. Therefore, the diagnosis should not rely only on the presence of apical ballooning but on the broader pattern of transient LV dysfunction extending beyond a single coronary territory, supported by clinical background, angiography, echocardiography, CMR, and follow-up recovery.

Recurrent and secondary TTS add another layer of complexity. Recurrence may occur with a similar or different ballooning pattern [15], while secondary TTS may develop during acute neurological disease, sepsis, respiratory failure, surgery, malignancy, or other critical illnesses [16]. In these settings, the cardiac syndrome may be overlooked because symptoms are masked by the underlying disease. Importantly, secondary TTS is often associated with worse outcomes than emotionally triggered TTS, reflecting both the severity of the precipitating illness and the higher burden of comorbidities [17,18].

## 3. Clinical Presentation and Mimics

For practical clinical purposes, TTS can be approached through several overlapping phenotypes, including emotionally triggered, physically triggered, neurologic, endocrine-triggered, MINOCA-like, recurrent, and high-risk or shock presentations. This phenotype-based approach is useful because each presentation has a different pattern of diagnostic uncertainty and a distinct set of differential diagnoses.

A clinically useful distinction is between emotionally triggered and physically triggered TTS [19]. Emotional TTS, the classical “broken heart syndrome”, is typically precipitated by grief, fear, anger, interpersonal conflict, or occasionally positive emotional events. It more commonly affects postmenopausal women and often presents with chest pain and an ACS-like phenotype [20]. Physical TTS occurs in the context of acute medical illness, surgery, trauma, respiratory failure, sepsis, neurological disorders, malignancy, or endocrine crises. These patients may present less typically, with dyspnea, shock, arrhythmias, or incidental ventricular dysfunction. Physical TTS is particularly important because it is often under-recognized and is generally associated with a higher complication burden and worse prognosis than emotionally triggered TTS [21].

TTS typically presents as an ACS with chest pain, dyspnea, syncope, palpitations, or cardiogenic shock. Electrocardiographic findings may include ST-segment elevation, ST-segment depression, T-wave inversion, QT interval prolongation, or non-specific repolarization abnormalities. Cardiac troponin is usually elevated, although the degree of elevation is often modest compared with the extent of ventricular dysfunction [22]. In contrast, natriuretic peptide levels are frequently markedly increased, reflecting acute ventricular wall stress [23]. This combination of clinical symptoms, ECG abnormalities, and biomarkers elevation explains why TTS is frequently indistinguishable from ACS at presentation.

Therefore, the most important diagnostic mimic is acute myocardial infarction. Patients with ST-segment elevation should be managed initially as STEMI until obstructive coronary artery occlusion is excluded. Similarly, patients presenting as NSTEMI require careful evaluation because TTS may coexist with CAD or may be triggered by an ACS [24,25]. However, the presence of coronary stenosis should not automatically exclude TTS; rather, clinicians should determine whether the coronary lesion explains the full extent and distribution of the wall-motion abnormality [26]. A mismatch between the coronary anatomy and the regional ventricular dysfunction should raise suspicion for TTS.

Myocarditis is another major mimic, particularly in patients with chest pain, troponin elevation, unobstructed coronary arteries, and regional wall-motion abnormalities [27]. Clinical clues such as viral prodrome, fever, inflammatory syndrome, younger age, or diffuse ECG abnormalities may suggest myocarditis. However, those features are not sufficiently specific, as even in endomyocardial biopsy secondary myocarditis-like changes have been described in TTS cases [28]. CMR is central in this distinction because myocarditis typically shows myocardial edema in acute phase and non-ischemic patterns of late gadolinium enhancement (LGE) after long time. TTS is characterized by reversible edema matching the dysfunctional segments, usually without irreversible necrosis or with only minimal transient LGE depending on imaging thresholds [29]. When uncertainty persists, follow-up imaging showing complete or near-complete recovery supports the diagnosis of TTS.

In this context, MINOCA should be considered a working diagnosis rather than a final diagnosis [30]. In patients presenting with ACS-like symptoms, troponin elevation, and non-obstructive coronary arteries, the underlying mechanism may include TTS, myocarditis, plaque disruption, coronary spasm, coronary embolism, or spontaneous coronary artery dissection [31]. Therefore, CMR, careful coronary review, and the selected use of intravascular imaging or vasomotor testing are important to avoid misclassification.

Endocrine-triggered TTS deserves specific attention, particularly pheochromocytoma-induced TTS [32]. In this setting, excessive catecholamine release provides a direct mechanistic link to myocardial stunning, microvascular dysfunction, and dynamic ventricular ballooning [33]. The clinical presentation may closely mimic ACS, myocarditis, hypertensive crisis, acute pulmonary edema, or fulminant cardiogenic shock. Clinical clues include paroxysmal hypertension, headache, sweating, palpitations, recurrent or unexplained TTS episodes, adrenal mass, and marked blood pressure variability [34]. Recognition is essential because definitive management requires the diagnosis and treatment of the catecholamine-secreting pheochromocytoma. In this case, beta-blockers should be avoided before adequate alpha-blockade because they may worsen hypertension through unopposed alpha-adrenergic stimulation [34]. Of note, LV recovery in pheochromocytoma-induced TTS is usually rapid [35].

Another clinically important presentation is the high-risk or shock phenotype of TTS [36]. These patients may present with cardiogenic shock, severe LV systolic dysfunction, RV involvement, significant mitral regurgitation, LVOTO, ventricular arrhythmias, or LV thrombus. This phenotype requires rapid differentiation from extensive AMI, fulminant myocarditis, pulmonary embolism, septic cardiomyopathy, and acute mechanical or valvular complications, because management differs substantially according to the underlying mechanism of shock.

Other important mimics include coronary vasospasm, spontaneous coronary artery dissection [37], microvascular angina, pulmonary embolism [38], aortic dissection [39], hypertrophic cardiomyopathy with dynamic obstruction [40], and neurogenic or critical illness-related myocardial dysfunction [41]. Neurological triggers, including subarachnoid hemorrhage [42], stroke [43], seizures [44], and psychiatric illness [45], are particularly relevant because they may produce catecholamine-mediated myocardial stunning with overlapping features. In critically ill patients, sepsis-related cardiomyopathy and stress cardiomyopathy may coexist or overlap, making their distinction difficult [46]. In such cases, the diagnostic goal is not simply to label syndrome but to exclude treatable coronary or structural causes and guide safe management. The main clinical phenotypes of TTS and their key differential diagnoses are summarized in Table 2.

## 4. Role of Cardiac Imaging

*Echocardiography*

Transthoracic echocardiography (TTE) is the first-line imaging investigation tool in TTS, allowing for the rapid assessment of LV and RV function, wall-motion phenotype, and acute complications. The typical finding is apical ballooning, caused by apical akinesia or dyskinesia with basal hyperkinesia, although mid-ventricular, basal, focal, and global phenotypes are increasingly recognized [9]. Importantly, the wall-motion abnormality usually extends beyond a single epicardial coronary territory. Echocardiography also identifies clinically relevant complications, including LVOTO, mitral regurgitation, RV involvement, and LV thrombus [49,50].

Advanced echocardiographic techniques, including contrast echocardiography, speckle-tracking strain, and coronary flow assessment [51], may improve visualization, quantify reversible myocardial dysfunction, and support differentiation from ACS in selected patients [51,52,53]. Speckle-tracking typically shows transient deformation abnormalities extending beyond a single vascular territory, while simplified approaches such as the TAKO tool have shown promising diagnostic accuracy for distinguishing TTS from LAD-related ACS [54].

*Cardiac magnetic resonance imaging (CMR)*

CMR is generally indicated when echocardiographic assessment is suboptimal, when the presentation is atypical, or when myocarditis, MINOCA, or infarction with non-obstructive coronary arteries remain in the differential diagnosis [9,47,55]. In the acute phase, CMR typically shows reversible myocardial edema involving the dysfunctional segments, with absent or minimal late gadolinium enhancement, supporting TTS rather than infarction or myocarditis [56]. It also provides accurate assessment of LV and RV morphology, detects LV thrombus or pericardial effusion, and may identify incomplete recovery during follow-up [57].

CMR feature-tracking and mapping techniques can detect subtle reversible myocardial injury and persistent functional abnormalities, although their routine diagnostic role remains limited [57]. RV involvement detected by CMR is associated with prolonged hospital stay and higher rates of adverse events and may be missed by TTE [52]. Because TTS may mimic ACS and coexist with CAD, CMR findings should be interpreted alongside coronary angiography or CTCA. In MINOCA, CMR is particularly valuable; a meta-analysis of 26 studies including 3624 patients showed diagnostic reclassification in 68% of cases and identified TTS in 10% [56].

*Invasive Coronary angiography*

**Figure 1:**
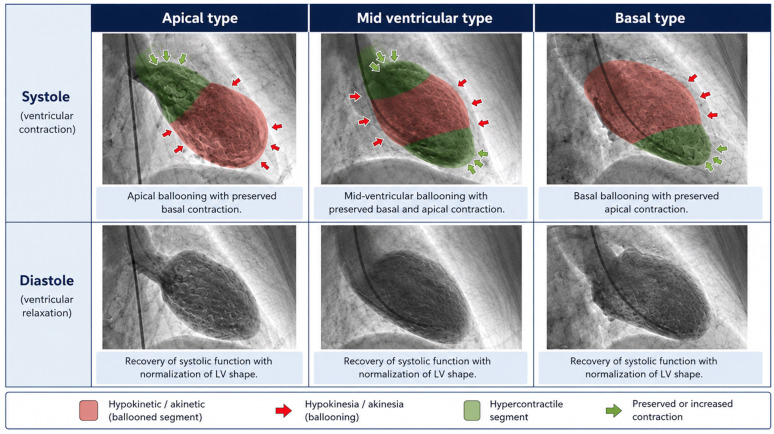
Angiographic patterns of Takotsubo syndrome. Left ventricular angiography during systole (**top row**) demonstrates regional wall-motion abnormalities in the three main variants: apical, mid-ventricular, and basal types. The affected segments are hypokinetic or akinetic (red), resulting in characteristic ballooning, while the non-affected segments are hypercontractile (green). During diastole (**bottom row**), normalization of ventricular function and shape is observed. LV: Left ventricle.

Because TTS frequently presents as ACS, invasive coronary angiography is recommended in patients with ST-segment elevation, high-risk ACS features, or hemodynamic instability to exclude culprit obstructive CAD [58]. Normal coronary arteries or non-obstructive CAD are typical, although stable concomitant CAD does not exclude TTS [58,59]. Left ventriculography can demonstrate regional wall-motion abnormalities extending beyond a single coronary territory and may reveal the characteristic ballooning phenotype, including apical, mid-ventricular, or basal patterns (Figure 1) [59,60]. Also, the “apical nipple sign,” which represents a small area of preserved contraction at the distal LV apex, can be seen in roughly one-third of TTS patients [9].

Complementary measurements performed during ICA include the intracoronary imaging methods such as intravascular ultrasound (IVUS) or optical coherence tomography (OCT), for ambiguous stenosis of coronary arteries. ICA may also provide functional information beyond the exclusion of obstructive CAD. In selected cases, invasive vasomotor testing and indices of microvascular resistance can demonstrate transient coronary microvascular dysfunction, supporting its proposed role in the pathophysiology of TTS [61,62]. The invasive hemodynamic assessment of the LV can be employed to measure the elevated LV end-diastolic pressure [48,63]. LVOT obstruction occurs in approximately 20% of individuals diagnosed with TTS, and its detection is important guiding further the therapeutic management [63].

*Computed tomography coronary angiography (CTCA)*

CCTA is a non-invasive imaging option, alternative to ICA, used to assess epicardial coronary arteries’ patency [58]. Beyond excluding CAD, CTCA can evaluate LV function, apical thrombi, and alternative diagnoses such as pulmonary embolism or aortic dissection, making it valuable in doubtful cases or when ICA is high-risk (e.g., septic shock, intracranial bleeding) or not immediately available [64,65,66]. Thereby, CCTA is increasingly recognized as an alternative in selected TTS, particularly those who are hemodynamically stable, with a circumferential ballooning pattern, and a low pre-test probability of CAD [52,58]. Recent stepwise non-invasive algorithms have suggested the combination of ECG, echocardiography and CCTA to confirm the typical TTS wall-motion pattern, while safely ruling out obstructive CAD. This approach is advantageous among elderly or high-risk patients in whom ICA would carry greater procedural risk [67,68,69]. Beyond luminal assessment, emerging CCTA techniques allow for the quantification of parameters such as extracellular volume fraction and detailed plaque characterization, which may provide additional insight into myocardial injury and concomitant risk for atherosclerotic plaque rupture in moderate stenosis. However, their prognostic value still needs validation in larger, prospective cohorts [70,71].

*Nuclear imaging*

Nuclear imaging is not part of routine diagnostic criteria but may provide mechanistic and prognostic information in selected patients. SPECT and PET techniques can assess myocardial perfusion, metabolism, microvascular function, and sympathetic innervation. TTS has been associated with perfusion–metabolism mismatch, impaired fatty acid or glucose metabolism, reduced ^123^I-MIBG uptake, and the delayed recovery of sympathetic innervation despite normalization of perfusion [72,73]. These combined perfusion–metabolic patterns with persistent abnormalities in glucose metabolism and sympathetic innervation can help diagnose TTS, particularly in patients presenting late after the triggering event [74], supporting the catecholamine-mediated pathophysiology, which differentiates TTS from AMI [75,76,77].

PET studies have also demonstrated the reversible impairment of myocardial blood flow and flow reserve, supporting coronary microvascular dysfunction as a potential mechanistic substrate [78,79,80,81,82]. These findings may be useful in late presentations or diagnostically uncertain cases, but limited availability, cost, radiation exposure, and a lack of standardized diagnostic thresholds restrict routine clinical use. Future AI-based integration of these multimodality data could improve diagnostic discrimination and risk stratification, but prospective external validation is required before clinical adoption [81,82,83].

*Biomarkers and Ancillary Tests*

In individuals with TTS, cardiac injury markers such as troponin, CK-MB, and myoglobin are usually elevated but generally to a lesser extent than in ACS, reflecting limited myocardial necrosis despite often extensive regional wall-motion abnormalities [84,85]. This mismatch between modest necrosis marker release and marked ventricular dysfunction may provide a useful diagnostic clue, although it cannot be used as a stand-alone criterion [86]. Natriuretic peptide-based ratios appear more diagnostically useful. BNP or NT-proBNP levels are typically disproportionately elevated compared with troponin or CK-MB in TTS, resulting in higher BNP/troponin, NT-proBNP/troponin, or BNP/CK-MB ratios than in ACS [87,88,89]. In a recent meta-analysis Couch et al. showed that troponin levels were significantly lower in TTS than in ACS patients, while natriuretic peptide levels were significantly higher [88]. Also, Rallidis et al. showed that an NT-proBNP/cTnT ratio > 7.5 on day 2 discriminated TTS from ACS with ~96% accuracy [90]. The similar diagnostic performance of BNP/Trop-I ratios is highlighted in a recent systematic review [91]. The ratio of natriuretic peptides and other markers of myocardial injury may be another finding to distinguish TTS. Randhawa et al. suggested that natriuretic peptide-based ratios, including BNP/CK-MB and NT-proBNP/myoglobin, may help distinguish TTS from AMI, with high specificity but variable sensitivity depending on the clinical setting and timing of sampling [23]. From the pathophysiology perspective, acute but reversible ventricular dysfunction and wall-stress in TTS drive robust natriuretic peptide release, whereas myocyte necrosis (and thus troponin release) is relatively limited compared with transmural infarction [91]. However, differences in assay types, sampling time points and cut-offs across studies have prevented the standardization of troponin/BNP ratios, and current guidelines call for further large, prospective, externally validated studies before routine implementation in diagnostic algorithms [91].

In patients with TTS, elevated systemic inflammatory markers were frequently observed on or close to admission, indicating a strong inflammatory response early in the disease course [92]. Circulating cytokines show distinct signatures: pro- and anti-inflammatory mediators such as Interleukin-6 (IL-6), Interleukin-10 (IL-10), Tumor necrosis factor-α (TNF-α), and others (e.g., IL-2, IL-4, IFN-γ) have been documented in these patients, supporting the activation of both innate and adaptive immune responses [93,94]. These inflammatory markers are increased in patients with TTS in the acute phase and remain elevated for several months [63]. IL-6 levels are elevated to a greater extent in patients with AMI compared to those with TTS, reflecting the larger extent of myocardial tissue damage in AMI [63]. In a prospective study of 55 TTS patients vs. matched controls, serum IL-6 was markedly higher in the acute phase and myocardial inflammation, evidenced by macrophage infiltration on ultrasmall superparamagnetic particles of iron oxide (USPIO) MRI, underscoring a link between systemic inflammation and myocardial tissue changes [94]. Importantly, residual inflammation at discharge, defined as a CRP > 19 mg/L, was associated in a large registry of 385 TTS patients with impaired left ventricular recovery and increased long-term cardiac mortality or heart failure hospitalization, pointing to the prognostic significance of persistent inflammation [95]. Moreover, a recent study highlighted that combining CRP measurement with conventional risk-stratification tools improves prognostic accuracy in TTS patients, supporting inflammation-based risk stratification as a promising adjunct [96].

Recently, attention has shifted toward novel biomarkers, including circulating catecholamines and microRNAs (miRNAs), to improve understanding, diagnosis, and risk stratification of TTS [97]. A study showed that serum catecholamine concentrations in these patients were significantly lower than levels observed in experimental TTS, challenging the traditional assumption that overt systemic catecholamine surges are universally observed in human TTS, and suggesting instead a possible role for local (e.g., myocardial or neuro-humoral) catecholamine effects or transient, rapidly cleared catecholamine spikes [62,97]. Regarding non-coding RNAs, a recent systematic review identified a panel of miRNAs (such as miR-1, miR-16, miR-26a, and miR-133a) whose circulating levels appear distinct in TTS compared with AMI, highlighting miRNAs as potentially sensitive and specific biomarkers for TTS [97,98]. Also, it seems that miRNAs implicated in TTS are involved in key pathophysiological processes underscoring their potential not only as diagnostic markers but also as indicators of disease mechanism or therapeutic targets [98].

Copeptin, a stable peptide fragment derived from the precursor of vasopressin, released early during stress, may enhance diagnostic accuracy when used with traditional markers [99,100]. It rises rapidly under stress, and it is usually lower in TTS than in STEMI. Notably, the copeptin/NT-proBNP ratio effectively separates TTS from AMI [85]. Integrated biomarker studies combining catecholamines, cytokines, glucocorticoid receptor expression and miRNA profiles propose that such multimodal biomarker signatures may improve discrimination of TTS from other acute cardiac conditions and enhance risk stratification, especially when traditional markers (troponin, BNP) and imaging are equivocal [101]. Despite their potential diagnostic value, most proposed biomarkers in TTS remain limited by important practical and methodological constraints. Natriuretic peptide-to-troponin ratios are attractive because they rely on widely available tests, but the proposed cut-offs vary across studies according to the assay type, the timing of blood sampling, renal function, age, heart failure status, and the comparator population. Similarly, copeptin, inflammatory markers, catecholamines, and microRNAs may provide mechanistic insight, but their clinical implementation is limited by cost, restricted availability, a lack of assay standardization, and insufficient external validation. MicroRNA panels, including miR-1, miR-16, miR-26a, and miR-133a, have been investigated because of their association with myocardial injury, inflammation, stress response pathways, and cardiomyocyte dysfunction; however, current studies are small and heterogeneous, and direct comparisons with established biomarkers such as high-sensitivity troponin and natriuretic peptides remain limited. Therefore, these emerging biomarkers should currently be considered investigational rather than routine diagnostic tools. A practical summary of the multimodality diagnostic work-up and its management implications is provided in Table 3.

## 5. Clinical Decision-Making and Management Implications

**Figure 2:**
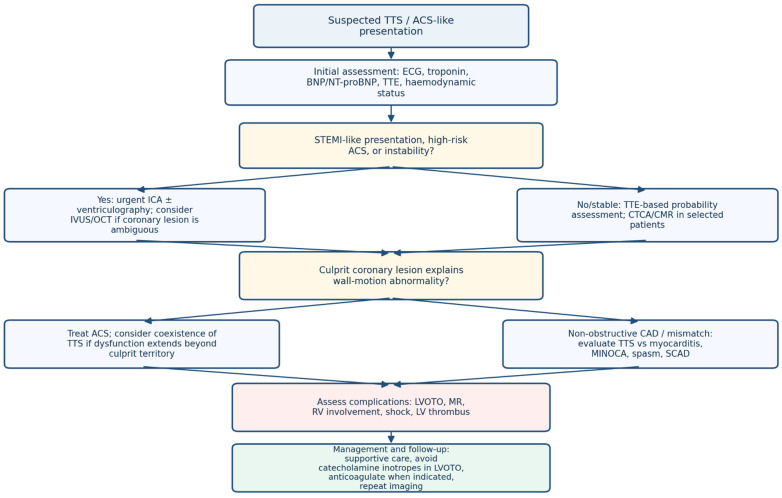
Proposed diagnostic and management algorithm for suspected Takotsubo syndrome. Author-proposed algorithm intended as a practical framework for acute diagnostic work-up and management. This pathway has not been externally validated and should complement, not replace, individualized clinical judgment and local protocols. ACS: acute coronary syndrome; BNP: B-type natriuretic peptide; CMR: cardiac magnetic resonance; CTCA: coronary computed tomography angiography; ECG: electrocardiogram; ICA: invasive coronary angiography; IVUS: intravascular ultrasound; LV: left ventricular; LVOTO: left ventricular outflow tract obstruction; MINOCA: myocardial infarction with non-obstructive coronary arteries; MR: mitral regurgitation; NT-proBNP: N-terminal pro-B-type natriuretic peptide; OCT: optical coherence tomography; RV: right ventricular; SCAD: spontaneous coronary artery dissection; STEMI: ST-segment elevation myocardial infarction; TTE: transthoracic echocardiography; and TTS: Takotsubo syndrome.

The clinical management of suspected TTS is strongly influenced by the initial differential diagnosis. Because the presentation frequently mimics ACS, patients should initially be treated according to ACS pathways until acute coronary occlusion has been excluded, particularly when ST-segment elevation, ongoing chest pain, hemodynamic instability, or high-risk ECG features are present. Urgent ICA remains the preferred diagnostic strategy in most unstable patients or in those with STEMI-like presentation [104]. In hemodynamically stable patients with a high probability of TTS and low probability of obstructive CAD, a non-invasive strategy incorporating echocardiography, CTCA and CMR may be considered, when does not delay possible ACS treatment [62]. Based on the available evidence and expert consensus, we propose a practical diagnostic and management framework for suspected TTS (Figure 2). This author-proposed algorithm has not been externally validated and should be interpreted as a clinical aid rather than a validated decision rule.

Once TTS is suspected or confirmed, management should be individualized according to hemodynamic status, ventricular function, and complications [105]. Supportive therapy is the cornerstone. In uncomplicated cases, beta-blockers, angiotensin-converting enzyme inhibitors or angiotensin receptor blockers and diuretics, may be considered depending on the clinical scenario, although robust randomized data are lacking [106]. Patients with significant LV dysfunction, apical akinesia, or visible thrombus require careful assessment for anticoagulation because of the risk of LV thrombus formation and systemic embolization [107].

A key management challenge is cardiogenic shock [108]. In TTS, shock may result from severe LV systolic dysfunction, right ventricular involvement, severe mitral regurgitation, LVOTO, or a combination of these mechanism. This distinction is clinically crucial. In patients with LVOTO, catecholamine inotropes and aggressive diuresis may worsen obstruction and hemodynamics [109]. In contrast, cautious beta-blockade, volume optimization, and the avoidance of vasodilators may be required. When shock occurs without LV outflow tract obstruction, mechanical circulatory support may be considered in selected patients [110]. The 2018 international consensus emphasizes that diagnostic work-up and management should be guided by the presence of complications, hemodynamic instability, and the need to differentiate TTS from ACS and myocarditis [58].

Risk stratification should not stop after the acute diagnosis [111]. Although TTS is reversible in many patients, it is not always benign. Adverse predictors include physical triggers, male sex, acute neurological or psychiatric disease, severe LV dysfunction, right ventricular involvement, cardiogenic shock, significant mitral regurgitation, LVOTO, elevated inflammatory markers, and incomplete recovery. The InterTAK Prognostic Score provides a practical tool for early risk assessment by combining clinical, ECG, biomarker, and LV functional variables associated with adverse in-hospital outcomes [112]. However, its limitations should be acknowledged. The score was derived from registry-based TTS populations and is mainly designed to estimate mortality risk rather than to provide diagnostic discrimination across specific anatomical subtypes. External validation remains less extensive than for widely used ACS risk scores, and data on its performance across apical, mid-ventricular, basal, focal, primary, and secondary TTS phenotypes are limited. Moreover, sensitivity and specificity thresholds are not uniformly established for all clinical settings. Therefore, the InterTAK Prognostic Score should complement, rather than replace, individualized clinical assessment, imaging-based risk evaluation, and careful follow-up.

Echocardiography is usually required to evaluate ventricular function, while CMR is valuable when recovery is incomplete, myocarditis remains possible, or tissue characterization is needed. Long-term care should also address precipitating factors, psychiatric or neurological comorbidities, recurrent symptoms, and cardiovascular risk factors [113]. Repeated echocardiography tests are adequate for LV function monitoring during follow-up.

## 6. Future Directions

Multimodality imaging will remain central, with echocardiography as the first-line tool and CMR, CTCA, nuclear imaging, and ICA used selectively to clarify coronary anatomy, myocardial edema, microvascular dysfunction, sympathetic abnormalities, and complications [52]. However, cost, availability, local expertise, scanner access, contrast exposure, and AI infrastructure may limit implementation in smaller hospitals or resource-constrained healthcare systems. Therefore, future pathways should define a minimum core work-up based on ECG, biomarkers, TTE, and coronary assessment when indicated, while reserving advanced modalities for diagnostically uncertain or high-risk cases.

AI may further improve diagnostic accuracy by integrating ECG, all imaging modalities, biomarkers, and clinical data [102]. In an echocardiography-based cohort study of 224 TTS and 224 AMI patients, a deep learning model achieved a mean AUC of 0.79 and accuracy of 74.8% in an independent validation set, outperforming cardiologists (mean AUC 0.71; accuracy 64.4%); in apical TTS versus LAD-related AMI, performance improved to AUC 0.84 and accuracy 78.6% [102]. ECG-based machine learning models have also shown promising discrimination, with reported ROC AUC values of 0.88 for TTS versus STEMI, 0.86 for TTS versus NSTEMI, and 0.85 for TTS versus suspected MI, although positive predictive values remain low and performance vary according to thresholds and case mix [103]. Additionally, explainable deep learning echocardiographic studies suggest that reduced atrioventricular-plane displacement and altered basal mechanics may represent discriminative imaging features in TTS [114], while machine learning protocols appear feasible to distinguish emotional versus physical TTS etiologies [115]. Nevertheless, these models remain investigational and require prospective external validation, calibration assessment, decision curve analysis, the reporting of sensitivity and specificity across TTS subtypes, and evidence that they improve clinical decisions before routine adoption.

Future research should also focus on validated biomarker panels, including natriuretic peptide-to-troponin ratios, inflammatory markers, catecholamines, microRNAs, and copeptin [116]. Standardized sampling, uniform diagnostic definitions, and longitudinal follow-up are needed.

### Limitations of This Review

This review has several limitations. First, it was designed as a narrative review rather than a systematic review or meta-analysis; therefore, study selection was not based on a formal protocol, and publication bias cannot be excluded. Second, much of the available evidence in TTS derives from observational registries, retrospective cohorts, small imaging studies, and expert consensus documents, while randomized data remain scarce. Third, diagnostic thresholds for biomarkers, imaging parameters, and AI-based tools vary substantially across studies, limiting direct comparison and generalizability. Finally, the rapidly evolving nature of AI, multimodality imaging, and biomarker research means that several emerging tools remain investigational and require prospective validation before routine clinical use.

## 7. Conclusions

TTS remains a diagnostic challenge because it closely mimics ACS, myocarditis, MINOCA, neurogenic myocardial injury, and critical illness-related cardiomyopathy. Although transient ventricular dysfunction is the defining feature, anatomical variants, heterogeneous triggers, and coexistence with CAD may complicate diagnosis. Contemporary criteria, clinical scores, biomarkers, and multimodality imaging have improved recognition, but no single test is definitive in all cases. Accurate diagnosis requires the integration of clinical assessments of neurological, psychiatric, and physical triggers, coronary anatomy, ventricular phenotype, tissue characterization, and follow-up LV recovery. Finally, structured post-discharge pathways, including repeat imaging, may improve risk stratification, detect recurrence, and support recovery.

## Figures and Tables

**Table 1:** Evolution of diagnostic criteria and diagnostic probability assessment in Takotsubo syndrome.

Score	Main Components	Strengths	Limitations	Practical Role
Mayo Clinic diagnostic criteria [4]	Transient LV dysfunction No culprit CAD/plaque rupture ECG/troponin changes Excludes myocarditis/pheochromocytoma	Simple Historical standard	Restrictive Under-recognizes CAD, atypical and secondary TTS	Historical framework Not sufficient as sole criterion today
Heart Failure Association/ESC diagnostic criteria [3]	Transient regional dysfunction Emotional/physical/neurological triggers RV and secondary forms recognized Recovery on follow-up	Broader real-world phenotype Includes secondary TTS	Requires clinical imaging integration Recovery may be delayed	Contemporary diagnostic and follow-up framework
InterTAK Diagnostic Score [8]	7 variables: sex, triggers, ECG, psychiatric/neurological disorders and QT prolongation	Bedside probability estimate Useful before definitive imaging	Does not replace urgent coronary assessment when ACS is likely	Early TTS-vs.-ACS triage tool
InterTAK Diagnostic Criteria [9]	LV/RV dysfunction Apical, mid-ventricular, basal, focal or global variants CAD may coexist Exclude myocarditis when suspected	Captures full TTS spectrum Accepts atypical variants	Mimics still need exclusion Challenging in critical illness	Preferred clinical and research classification

ACS: acute coronary syndrome; CAD: coronary artery disease; ECG: electrocardiogram; ESC: European Society of Cardiology; LV: left ventricular; RV: right ventricular; and TTS: Takotsubo syndrome [8]. This score is particularly useful in the emergency setting and has been validated in many cohorts worldwide [10,11]. The 2018 International Expert Consensus documents remain among the most important references for diagnostic criteria, work-up, prognosis, and management [9].

**Table 2:** Main clinical phenotypes of Takotsubo syndrome and key differential diagnoses.

Clinical Phenotype	Typical Presentation	Key Clinical or Imaging Clues	Main Differential Diagnoses
Emotional TTS [19,20,21]	Emotional stress, grief, fear, anger, interpersonal conflict, or positive emotional events; commonly postmenopausal women	ACS-like chest pain; modest troponin rise; marked NPs elevation; usually apical ballooning, although other phenotypes may occur	STEMI/NSTEMI; coronary vasospasm; myocarditis; SCAD
Physical TTS [19,20,21]	Acute medical illness, surgery, trauma, respiratory failure, sepsis, malignancy, or severe systemic stress	Dyspnea, shock, arrhythmias, or incidental LV dysfunction; biomarkers influenced by underlying illness; complications more frequent	ACS; sepsis-related cardiomyopathy; myocarditis; pulmonary embolism
MINOCA-like TTS [47,48]	ACS-like presentation with troponin elevation and non-obstructive coronary arteries	Wall-motion abnormality beyond a single coronary territory; CMR supports TTS when edema is present without infarct-like necrosis	Myocarditis; plaque disruption; coronary spasm; coronary embolism; SCAD
High-risk/shock phenotype [36]	Cardiogenic shock, severe LV dysfunction, RV involvement, severe MR, LVOTO, or ventricular arrhythmias	Hemodynamic instability; need to distinguish pump failure from LVOTO; possible LV thrombus or biventricular involvement	Extensive AMI; fulminant myocarditis; pulmonary embolism; septic cardiomyopathy; acute valvular complication
Neurologic/neurogenic TTS [40,41,42,43,44]	Subarachnoid hemorrhage, stroke, seizures, intracranial bleeding, or acute neurologic injury	QT prolongation; troponin/NP elevation; LV dysfunction in the setting of acute neurologic disease; basal or global patterns may occur	Neurogenic stunned myocardium; ACS; myocarditis; stress-related critical illness cardiomyopathy
Endocrine-triggered/pheochromocytoma-related TTS [32,33]	Paroxysmal hypertension, headache, sweating, palpitations, blood pressure lability, adrenal mass, or recurrent unexplained TTS	Catecholamine excess; basal/reverse or atypical ballooning more frequent, but any TTS pattern may occur; rapid LV recovery possible	ACS; myocarditis; hypertensive emergency; catecholamine-induced cardiomyopathy
Recurrent or trigger-negative TTS [9]	Previous TTS episode or no clear emotional/physical trigger	Similar or different ballooning pattern compared with prior episode; requires search for occult triggers	ACS; myocarditis; pheochromocytoma; vasospasm; occult neurologic or systemic trigger

ACS: acute coronary syndrome; AMI: acute myocardial infarction; CMR: cardiac magnetic resonance; LV: left ventricular; LVOTO: left ventricular outflow tract obstruction; MINOCA: myocardial infarction with non-obstructive coronary arteries; MR: mitral regurgitation; NPs: natriuretic peptides; NSTEMI: non-ST-segment elevation myocardial infarction; RV: right ventricular; SCAD: spontaneous coronary artery dissection; STEMI: ST-segment elevation myocardial infarction; and TTS: Takotsubo syndrome.

**Table 3:** Multimodality diagnostic work-up and management implications.

Modality/Test	Diagnostic Contribution	Typical TTS Finding	Main Limitations	Management Implication
ECG [9,67]	First-line in ACS-like presentation	STE/depression, T-wave inversion, QT prolongation	Non-specific; no reliable ACS/TTS distinction	Start ACS pathway when clinically indicated
Troponin/CK-MB [22]	Confirms myocardial injury	Modest rise despite marked LV dysfunction	Overlap with ACS/myocarditis	Severity clue; not stand-alone diagnosis
BNP/NT-proBNP [23,90]	Reflects acute wall stress	Disproportionately high vs. troponin	Affected by age, renal function, HF and AF	High BNP/troponin ratio supports TTS contextually
Inflammatory biomarkers [92,93]	Assess inflammation and prognosis	CRP/leukocytes may be elevated Persistent inflammation may predict worse recovery	Non-specific; affected by infection/comorbidity	May guide risk stratification/follow-up intensity
TTE [46,49,50]	First-line imaging; complications	Apical/mid/basal/focal/global dysfunction beyond one territory	Image quality; limited tissue characterization	Detects LVOT obstruction, MR, RV involvement and thrombus
Speckle-tracking echocardiography [51,52,53]	Quantifies deformation	Reversible strain impairment beyond one vascular territory	Availability and vendor variability	Differentiation from ACS; recovery monitoring
ICA/left ventriculography [69,70,71,74]	Excludes culprit CAD; shows ballooning	Non-obstructive/stable CAD; ventriculographic ballooning	Invasive; contrast/radiation	Essential in STEMI-like or high-risk cases
IVUS/OCT [75,76]	Clarifies ambiguous coronary lesions	No plaque rupture/erosion in isolated TTS	Invasive; selective use	Useful when CAD/SCAD remains uncertain
CMR [47,56]	Tissue characterization	Edema in dysfunctional segments; absent/minimal LGE; recovery	Availability, timing, contraindications	Distinguishes myocarditis/infarction; detects thrombus/RV involvement
CTCA [71]	Non-invasive coronary/alternative diagnosis assessment	No obstructive culprit CAD; may assess LV function	Not ideal if unstable/heavy calcification; contrast	Option in stable, selected low-intermediate CAD probability
Nuclear imaging/PET/MIBG [72,73]	Perfusion, metabolism, innervation	Perfusion–metabolism mismatch; impaired MIBG uptake	Limited availability; not routine criteria	Selected late/mechanistic/prognostic assessment
AI-based ECG/imaging tools [102,103]	Pattern recognition and data integration	Subtle ECG/wall-motion signatures	Needs external validation and workflow integration	Promising adjunct; not stand-alone

ACS: acute coronary syndrome; AF: atrial fibrillation; AI: artificial intelligence; BNP: B-type natriuretic peptide; CAD: coronary artery disease; CK-MB: creatine kinase-myocardial band; CMR: cardiac magnetic resonance; CRP: C-reactive protein; CTCA: coronary computed tomography angiography; ECG: electrocardiogram; HF: heart failure; ICA: invasive coronary angiography; IVUS: intravascular ultrasound; LGE: late gadolinium enhancement; LV: left ventricular; MIBG: metaiodobenzylguanidine; MR: mitral regurgitation; NP: natriuretic peptide; NT-proBNP: N-terminal pro-B-type natriuretic peptide; OCT: optical coherence tomography; PET: positron emission tomography; RV: right ventricular; SCAD: spontaneous coronary artery dissection; STE: ST-segment elevation; STEMI: ST-segment elevation myocardial infarction; TTE: transthoracic echocardiography; TTS: Takotsubo syndrome; and WMA: wall-motion abnormality.

## Data Availability

No new data were created or analyzed in this study.
